# Possible Role of Adenosine in COVID-19 Pathogenesis and Therapeutic Opportunities

**DOI:** 10.3389/fphar.2020.594487

**Published:** 2020-11-26

**Authors:** Jonathan D. Geiger, Nabab Khan, Madhuvika Murugan, Detlev Boison

**Affiliations:** ^1^ Department of Biomedical Sciences, University of North Dakota School of Medicine and Health Sciences, Grand Forks, ND, United States; ^2^ Department of Neurosurgery, Robert Wood Johnson Medical School, Rutgers University, Piscataway, NJ, United States; ^3^ Rutgers Neurosurgery H.O.P.E. Center, Department of Neurosurgery, Rutgers University, New Brunswick, NJ, United States

**Keywords:** coronavirus, COVID, acute lung injury, adenosine, adenosine deaminase, adenosine kinase

## Abstract

The outbreak of the novel coronavirus disease 2019 (COVID-19) caused by Severe Acute Respiratory Syndrome CoronaVirus-2 (SARS-CoV-2) requires urgent clinical interventions. Crucial clinical needs are: 1) prevention of infection and spread of the virus within lung epithelia and between people, 2) attenuation of excessive lung injury in Advanced Respiratory Distress Syndrome, which develops during the end stage of the disease, and 3) prevention of thrombosis associated with SARS-CoV-2 infection. Adenosine and the key adenosine regulators adenosine deaminase (ADA), adenosine kinase (ADK), and equilibrative nucleoside transporter 1 may play a role in COVID-19 pathogenesis. Here, we highlight 1) the non-enzymatic role of ADA by which it might out-compete the virus (SARS-CoV-2) for binding to the CD26 receptor, 2) the enzymatic roles of ADK and ADA to increase adenosine levels and ameliorate Advanced Respiratory Distress Syndrome, and 3) inhibition of adenosine transporters to reduce platelet activation, thrombosis and improve COVID-19 outcomes. Depending on the stage of exposure to and infection by SARS-CoV-2, enhancing adenosine levels by targeting key adenosine regulators such as ADA, ADK and equilibrative nucleoside transporter 1 might find therapeutic use against COVID-19 and warrants further investigation.

## Introduction

Coronavirus disease 2019 (COVID-19) is a global pandemic caused by Severe Acute Respiratory Syndrome Coronavirus-2 (SARS-CoV-2) (Guo et al., 2020). This highly infectious virus has spread globally with rapid and deadly consequences, and COVID-19 has overwhelmed health care systems. Accordingly, COVID-19 has focused attention not only on mechanisms by which the disease-causing coronavirus enters and infects the host but also on new treatment strategies. Given the steep rise in COVID-related deaths, developing novel therapies, vaccines and/or drugs, while important and of the highest priority, is expensive and time-consuming. Hence, re-evaluating and/or repositioning existing drugs for treatment of COVID-19 is the best immediate strategy to counter this pandemic (Kupferschmidt and Cohen, 2020; Moore et al., 2020). Clearly, therapeutic strategies are needed immediately to decrease viral spread, significantly attenuate acute respiratory distress, and reduce mortality and morbidities associated with COVID-19. Moreover, these strategies might reduce the tragic strain on health care workers and key hospital resources (Emanuel et al., 2020) as there are over 38.8 million cases world-wide and the full extent of the pandemic is still unknown.

**FIGURE 1:**
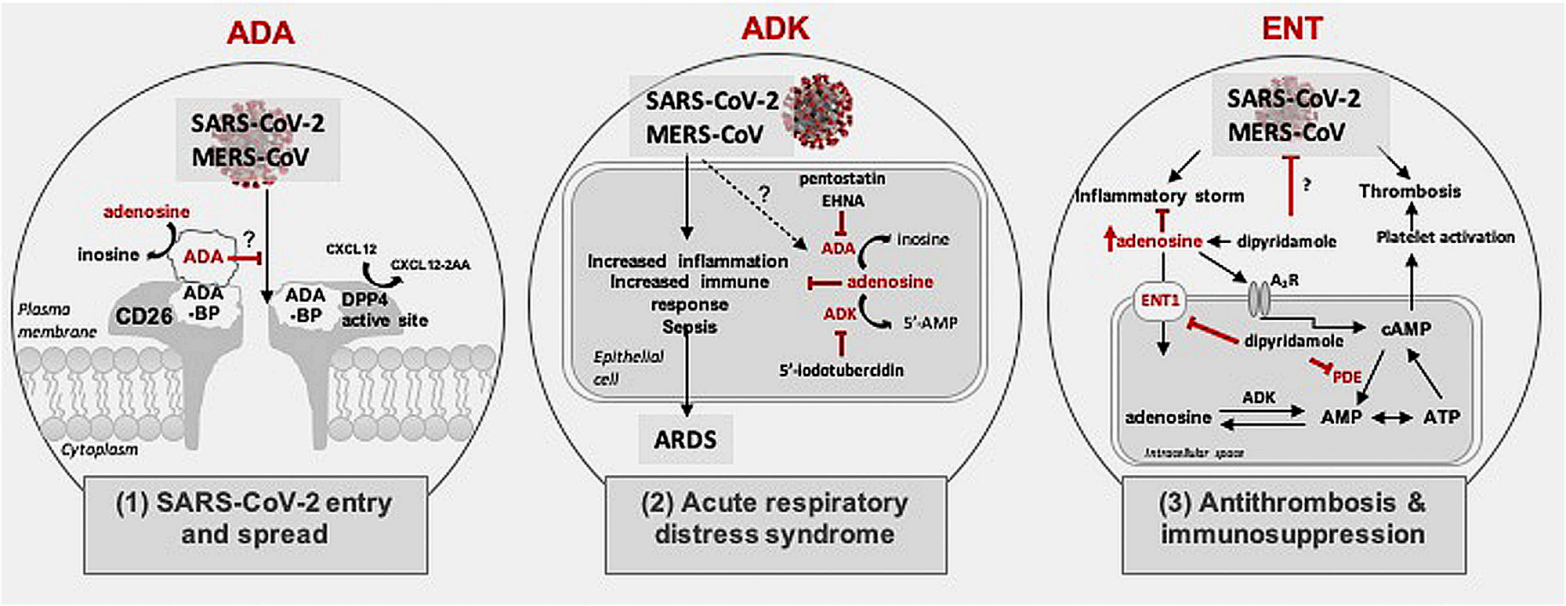
Possible role of adenosine in coronavirus pathogenesis. Schematic shows three mechanisms possibly regulated by adenosine in SARS-CoV-2 and MERS-CoV pathogenesis: 1) Role in SARS-CoV2 and MERS-CoV entry and spread: Schematic shows possible binding of SARS-CoV-2 glycoprotein to the CD26/Middle East respiratory syndrome coronavirus (MERS-CoV) receptor for cellular entry. The CD26 receptor houses a dipeptidyl peptidase 4 (DPP4) active site and an adenosine deaminase (ADA) binding protein (ADA-BP). ADA catalyzes the deamination of adenosine to inosine and competes with MERS-CoV for binding to ADA-BP. 2) Role in acute respiratory distress syndrome: Schematic shows adenosine level regulation via ADA and adenosine kinase (ADK). ADA and ADK inhibitors increase levels of extracellular adenosine, which in turn attenuates immune responses, inflammation, sepsis, and acute respiratory distress syndrome. 3) Role in thrombosis and inflammation: Schematic shows role of adenosine in thrombosis and inflammation. Inhibition of adenosine transport by targeting equilibrative nucleoside transporters type 1 (ENT1) using dipyridamole can enhance adenosine levels and may ameliorate inflammatory processes associated with coronavirus infection. Further, dipyridamole might prevent thrombosis via inhibiting phosphodiesterase (PDE) or directly act on SARS-CoV-2 through its antiviral properties.

Although the pathophysiology of COVID-19 remains largely enigmatic, recent studies identified similarities with Middle East Respiratory Syndrome CoronaVirus (MERS-CoV) and SARS-CoV (Huang et al., 2020; Li et al., 2020a; Ou et al., 2020). Based on published findings about binding partners for these two viruses as well as published findings from others and us, targeting the purine nucleoside adenosine and regulators of adenosine levels might yield new insight into COVID-19 pathogenesis as well as new therapeutic options for early and/or later stages of COVID-19 disease pathogenesis (Figure 1). Elevated tissue tone of adenosine is known to exert immunosuppressive action through activation of adenosine receptors (A2A and A2B). Hence targeting adenosinergic pathway components and adenosine A2A receptor signaling is predicted to be an effective treatment strategy for COVID-19 (Abouelkhair, 2020). In this review, we highlight putative roles of three key adenosine regulators; adenosine deaminase (ADA), adenosine kinase (ADK) and equilibrative nucleoside transporter-1 (ENT1). Further, we discuss potential therapeutic opportunities based on interference with 1) cell adhesion/virulence, 2) inflammatory and immune processes associated with acute severe respiratory distress syndrome (ARDS), and 3) hypercoagulation/thrombosis.

### Targeting Adenosine Deaminase to Potentially Prevent Severe Acute Respiratory Syndrome Coronavirus-2 Cell Adhesion and Virulence

Preventing SARS-CoV-2 entry into lung epithelial cells and reducing the spread of infection may be possible by targeting the non-enzymatic role of ADA. MERS-CoV uses the ADA-binding protein dipeptidyl peptidase 4 (DPP4) active sites on CD26 and to a lesser extent angiotensin I converting enzyme 2 (ACE2) as mechanisms for coronavirus binding and infectivity (Rasmussen et al., 2003; Song et al., 2014; van Doremalen et al., 2014; Ou et al., 2020; Qi et al., 2020; Walls et al., 2020). In contrast, recent studies implicate ACE2 as the predominant receptor mechanism for SARS-CoV-2 virus entry and endocytosis (Hoffmann et al., 2020; Ou et al., 2020; Walls et al., 2020). However, the expression levels of ACE2 are low, particularly in the lung. Further, a model for SARS-CoV-2 spike protein forming docked complexes with DPP4 has been proposed (Strollo and Pozzilli, 2020; Eleftheriou et al., 2020; Li et al., 2020b). Hence, SARS-CoV-2 may use co-receptors/auxiliary proteins such as CD26-resident co-receptors including DPP4 to facilitate virus entry (Huang et al., 2020). CD26/DPP4 is a well-known regulator of immune functions and these actions are initiated by cleavage of penultimate N-terminal L-proline or L-alanine amino acids from polypeptides (Morimoto and Schlossman, 1998). DPP4 inhibitors are widely used clinically and have been shown to enhance clinical outcomes and reduce mortality in diabetes mellitus patients with COVID-19 (Mirani et al., 2020; Rhee et al., 2020; Solerte et al., 2020). However, this improvement may not be due to antiviral actions of these drugs but rather immune modulation and/or decreased inflammatory responses because such inhibitors were unable to block MERS-CoV infection (Strollo and Pozzilli, 2020). These reports provide preliminary evidence that treatment with Sitagliptin, a DPP-4 inhibitor, reduced mortality in patients with type 2 diabetes hospitalized for COVID-19. Given the relationship between DPP-4 and MERS-CoV, it is plausible that the protective effects of DPP-4 inhibitors are mediated by competing with the docking process of SARS-CoV-2 to DPP-4/CD26 at membranes of relevant cells and thereby preventing viral entry (Lu et al., 2013; Raj et al., 2014). However, this mechanism has been contested and alternative mechanism of action for DPP-4 inhibitors have been suggested (Nauck and Meier, 2020).

Infection with MERS-CoV is initiated by the binding of MERS-CoV spike protein to DPP4; a process that causes virus-cell fusion (Lu et al., 2013; Barlan et al., 2014; van Doremalen et al., 2014). Genetic variations in DPP4 block interactions between MERS-CoV spike protein and those variations cause species barriers to infection (Letko et al., 2018). Humans and frugivorous bats express DPP4 in respiratory and intestinal tract epithelial cells and such findings raise the possibility that transmission of MERS-like CoVs might occur via respiratory and fecal-oral routes (Widagdo et al., 2017). Tragically, people with co-morbid conditions appear to be especially at risk for increased mortality; levels of CD26 mRNA and protein are elevated in people who smoke tobacco and/or live with chronic obstructive pulmonary disease (COPD) (Seys et al., 2018). Similar to MERS-CoV, SARS-CoV-2 may interact with DPP4. However, unlike MERS-CoV, SARS-CoV-2 is highly infectious, causes ARDS, and has been associated with high rates of lethality (Vankadari and Wilce, 2020; Wu et al., 2020).

In addition to the DPP4 active site, the CD26 receptor also contains ADA binding protein (ADA-BP) (Gorrell et al., 2001). ADA is mainly an intracellular cytosolic adenosine-metabolizing enzyme, but depending on the type of cell, it can also be expressed as an ectoenzyme; ecto-ADA binds to ADA-BP and once bound, ADA catalyzes the deamination of adenosine to inosine (Figure 1). Both endo- and ecto-ADA function to help regulate levels of adenosine. This enzymatic process helps to regulate and fine-tune the anti-inflammatory and immunosuppressive roles of adenosine. Of most direct relevance to the possible use of ADA against early infection by SARS-CoV-2 are findings that MERS-CoV entry and infection were prevented with exogenous ADA outcompeting MERS-CoV binding to DPP4 (Raj et al., 2014). This possible therapeutic approach is possible because PEGylated ADA is an FDA-approved drug (Pegademase bovine) for use in people with severe combined immunodeficiency disease and recombinant ADA has been proposed as an effective adjuvant with HIV-1 vaccines (Tardif et al., 2017; Tardif et al., 2019). Although some side effects including hemolytic anemia, autoimmune hemolytic anemia and thrombocytopenia have been noted following long-term use of PEGylated ADA, relatively minor adverse events have been noted with short-term use (Booth and Gaspar, 2009). These findings raise the possibility of targeting ADA to control SARS-CoV2 binding to CD26 receptors and preventing the cellular entry of the virus and preventing the spread of infection within the lungs. We propose the use of PEGylated ADA to be used post-exposure to shorten the duration of both symptoms and viral spreading following COVID-19 infection. Most antivirals have been shown to effectively limit viral replication when administered within a specific time-frame following exposure. For instance, oseltamivir, the antiviral against influenza, is most effective if initiated <48 h after illness onset (McLean et al., 2015). Hence, the temporal window for maximum antiviral efficacy of PEGylated ADA needs to be determined and taken into consideration for optimal clinical outcomes.

### Targeting Enzymatic Roles of Adenosine Deaminase and Adenosine Kinase To Ameliorate Acute Severe Respiratory Distress Syndrome Symptoms

The enzymatic roles of ADK and ADA might be targeted to ameliorate ARDS that often is present in patients with severe COVID-19. Clinically, SARS-CoV-2 infection is associated with a wide range of clinical manifestations ranging from asymptomatic to severe. The severe cases present with pneumonia, which can progress to ARDS (Fung et al., 2020). In the first case study conducted in Wuhan, China, 84 out of 201 patients (41.8%) developed ARDS, and of those 44 (52.4%) died, making ARDS the leading cause of death among patients with COVID-19 (Wu et al., 2020). Some evidence suggests that treatments with the steroids methylprednisolone or dexamethasone may be beneficial to COVID-19 patients who develop ARDS (Wu et al., 2020). However, the continuing steep rise in COVID-related deaths, reaching close to 1.1 million worldwide, reflects the need for treatment options to ameliorate ARDS and to prevent death.

Adenosine is protective against a wide variety of inflammatory events associated with acute and chronic neurodegenerative disorders (Kohler et al., 2007; Morote-Garcia et al., 2009). Importantly, adenosine can protect against ARDS (Morote-Garcia et al., 2013; Kohler et al., 2016) and acute/chronic lung injury in preclinical mouse models (Kohler et al., 2016). Adenosine levels are regulated by a close interplay of anabolic and catabolic purine enzymes, nucleoside transporters, and adenosine release mechanisms (Geiger and Nagy, 1990; Geiger et al., 1997; Boison, 2013). Of the involved metabolic enzymes, ADA and ADK appear to be most prominent (Geiger and Nagy, 1990; Boison, 2013). This raises the possibility of targeting ADA and ADK for prevention and/or treatment of ARDS during later phases of COVID-19 (Figure 1). In support of this notion, we have previously demonstrated that transgenic reduction or pharmacological inhibition of ADK attenuates acute lung injury in mice (Kohler et al., 2016).

ADA is a low affinity high capacity enzyme for the metabolism of adenosine to inosine, becoming relevant when and where adenosine levels are high. Hence, inhibiting ADA to augment protective adenosine might be beneficial to improve clinical outcomes in COVID-19 patients. It is important to note that, while immunosuppression by adenosine can hinder the elimination of the virus, and control of viral replication during the early phase of infection (Passos et al., 2018), this might be beneficial in chronic stages of ARDS which is characterized by hyper-inflammation. However, the possible beneficial effects of immunosuppression and anti-inflammation should be carefully weighed up against the potential for deleterious impairment of anti-viral immunity (Ritchie and Singanayagam, 2020). Interestingly, deamination of adenosine by ADA counteracts the negative effects of adenosine in immune cells, boosting the immune response. To this end, the FDA approved ADA inhibitor pentostatin (2′-deoxycoformycin) may prove to be a promising candidate. Pentostatin is an inhibitor of the enzymatic actions of ADA and used clinically for the treatment of patients with Hairy Cell Leukemia (Kane et al., 1992). Because it is a highly potent and tightly binding inhibitor of ADA, it is infused into patients over a 20–30 min period and the effects last for a few weeks (Trincavelli, 2013). Pentostatin-induced elevation of levels of adenosine has immunosuppressive actions (Tardif et al., 2019) and might be beneficial in late stage ARDS. EHNA (erythro-9-(2-hydroxy-3-nonyl)adenine hydrochloride) is another ADA inhibitor that not only acts as a competitive inhibitor of ADA enzymatic activity, but may also influence non-enzymatic functions of ADA, such as binding to adenosine receptors and DPP4 (Gracia et al., 2013); although not used clinically it has been noted to have potent anticancer effects against malignant pleural mesothelioma (Nakajima et al., 2015).

ADK is the key regulator of adenosine under baseline conditions and catalyzes the metabolism of adenosine to 5′-adenosine monophosphate (Boison, 2013). In relation to ARDS, inhibition of ADK by 5′-iodotubercidin increased extracellular adenosine levels *in vitro*, diminished the transmigration of neutrophils, and improved the epithelial barrier function. Inhibition of ADK *in vivo* showed protective properties and reduced the extent of pulmonary inflammation during acute lung injury in a mouse model of lipopolysaccharide inhalation (Kohler et al., 2016). ADK inhibitors are potent anti-inflammatory agents, they may also be of therapeutic benefit in septic shock caused by COVID-19 (Firestein et al., 1994). ADK inhibitors were previously considered in pre-clinical models for a wide variety of conditions including epilepsy, pain, and chronic inflammatory diseases such as rheumatoid arthritis (Boison, 2013; Jarvis, 2019). Despite the success in preclinical models, there are no FDA-approved ADK inhibitors due to concerns associated with the intended long-term use of those agents, which include liver toxicity (Boison et al., 2002) and the occurrence of brain hemorrhage in some of the preclinical studies (McGaraughty et al., 2005). Inhibitors of ADK such as 4-(N-phenylamino)-5-phenyl-7-(5-deoxyribofuranosyl)pyrrolo[2,3-day]pyrimidine (GP683) and ABT-702 were primarily developed for chronic treatment paradigms such as for neuropathic pain and epilepsy and were abandoned due to toxicity in long-term usage (Boison, 2013). A novel class of stereoselective inhibitors targeting human ADK are under development (Toti et al., 2016) and might find use for acute/short-term use to attenuate life-threatening ARDS.

### Targeting Equilibrative Nucleoside Transporter-1 To Reduce Thrombosis and Heightened Immune Response

Adenosine transport might also be targeted to enhance adenosine levels and reduce platelet activation, decrease thrombosis, and attenuate inflammation to improve COVID-19 outcomes. Although ARDS still remains the predominant cause of mortality in COVID-19 patients (Wu et al., 2020), a substantial number of deaths due to coagulation abnormalities and thrombosis have been reported (Guan et al., 2020; Tang et al., 2020a). More importantly, coagulopathy in COVID-19 patients is associated with an increased risk of death (Tang et al., 2020b). The coagulation changes associated with COVID-19 suggests the presence of a hypercoagulable state as indicated by increased D-dimer concentration, a relatively modest decrease in platelet count, and a prolongation of the prothrombin time (Levi et al., 2020). Against this, dipyridamole might find use in COVID-19 patients. Dipyridamole is an ENT-1 inhibitor and an FDA approved drug indicated as an adjunct to anticoagulants in the prevention of postoperative thromboembolic complications of cardiac valve replacement (Taguchi et al., 1975). In fact, a recent study of twelve COVID-19 patients with prophylactic anti-coagulation therapy supplemented with dipyridamole showed significantly increased platelet and lymphocyte counts and decreased D-dimer levels in comparison to control patients (Liu et al., 2020).

The anticoagulant property of dipyridamole occurs via two main mechanisms (Figure 1). First, as an inhibitor of equilibrative nucleoside transporters (ENTs), dipyridamole prevents the intracellular transport of adenosine. The resultant excess adenosine exerts its effects through the activation of G-protein coupled receptors. In platelets, binding of adenosine to A_2_ receptor subtypes (A_2A_ or A_2B_) leads to the consequent elevation of intracellular cyclic adenosine monophosphate (cAMP), an inhibitor of platelet activation (Johnston-Cox and Ravid, 2011). Second, dipyridamole acts as a pan-phosodiesterase inhibitor, preventing the degradation of cAMP and thereby influencing anti-thrombosis (Gresele et al., 2011).

In chronic stages of COVID-19, increased concentrations of proinflammatory cytokines, such as tumor necrosis factor-α (TNF-α) and interleukins (IL), including IL-1 and IL-6 have been reported (Huang et al., 2020). In a subset of patients most severely affected by COVID-19, a cytokine storm profile is observed, characterized by high concentrations of proinflammatory cytokines and chemokines (Mehta et al., 2020). Hence, during the chronic stage of COVID-19, the anti-inflammatory role of elevated adenosine triggered by dipyridamole might be beneficial in ameliorating tissue inflammation. In addition to regulating adenosine levels, dipyridamole is also thought to have broad spectrum antiviral properties particularly efficacious against upper respiratory tract infections (Hu and Wang, 1995, Xie, 2010, Mehta et al., 2020). In view of the hypercoagulable state of patients with severe COVID-19, the increased risk of thrombosis and improved outcomes in cases with prophylactic treatment, a mandated anti-thrombotic treatment has been recommended in the absence of medical contraindications (Levi et al., 2020). The multifaceted functions and the success of dipyridamole adjunctive therapy in a cohort of COVID-19 patients (Liu et al., 2020) suggests that it might be an ideal candidate for prophylactic anti-thrombosis therapy.

Drugs that affect adenosine-related mechanisms might find use against SARS-CoV-2 infection and clinical features of COVID-19. However, in-silico and clinically-relevant animal models are needed to confirm the benefits of targeting adenosine metabolism in the context of COVID-19 infections. *In vitro* models such as human airway epithelial cells and lung organoids generated from human embryonic stem cells have been shown to be beneficial for investigating COVID-19 and testing therapeutic candidates (Han et al., 2020; Zhu et al., 2020). Detailed reviews of successful *in vitro* and preclinical animal models for COVID-19 basic research have been recently elucidated (Leist et al., 2020; Takayama, 2020). Hence, the immediate next steps would be to test these putative adenosine-based therapeutic candidates in these models. Further follow-up with randomized controlled trials are needed to test the effectiveness, mechanisms and safety of these compounds in improving the clinical outcomes of COVID-19.

## Data Availability Statement

The original contributions presented in the study are included in the article/supplementary material, further inquiries can be directed to the corresponding author.

## Author Contributions

All authors contributed equally to this work.

## Funding

JG gratefully acknowledges research funding support provided by the NIH; P30GM100329, U54GM115458, R01MH100972, R01MH105329, R01MH119000, 2R01NS065957, and 2R01DA032444. DB gratefully acknowledges research funding support provided by the NIH (NS065957, NS103740) and Citizens United for Research in Epilepsy (DB, CURE Catalyst Award).

## Conflict of Interest

The authors declare that the research was conducted in the absence of any commercial or financial relationships that could be construed as a potential conflict of interest.
